# Differential Effects of Thiazolidinediones on Adipocyte Growth and Recruitment in Zucker Fatty Rats

**DOI:** 10.1371/journal.pone.0008196

**Published:** 2009-12-24

**Authors:** Jennifer MacKellar, Samuel W. Cushman, Vipul Periwal

**Affiliations:** 1 Diabetes Branch, National Institute of Diabetes and Digestive and Kidney Diseases, National Institutes of Health, Bethesda, Maryland, United States of America; 2 Laboratory of Biological Modeling, National Institute of Diabetes and Digestive and Kidney Diseases, National Institutes of Health, Bethesda, Maryland, United States of America; Institute of Preventive Medicine, Denmark

## Abstract

**Background:**

Adipose tissue grows by two mechanisms: hyperplasia (cell number increase) and hypertrophy (cell size increase). Thiazolidinediones are insulin-sensitizing peroxisome proliferator-activated receptor gamma agonists that are known to affect the morphology of adipose tissue.

**Methodology:**

In this study, adipose cell-size probability distributions were measured in six Zucker fa/fa rats over a period of 24 days, from four weeks of age, using micro-biopsies to obtain subcutaneous (inguinal) fat tissue from the animals. Three of the rats were gavaged daily with rosiglitazone, a thiazolidinedione, and three served as controls. These longitudinal probability distributions were analyzed to obtain the rate of increase in cell-size diameter in rosiglitazone-treated animals, and the hyperplasia induced by treatment quantitatively.

**Conclusions:**

We found that treatment leads to hypertrophy that leads to an approximately linear rate of cell diameter increase (2 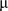
m/day), and that the hyperplasia evident in treated animals occurs largely within the first eight days of treatment. The availability of additional lipid storage due to treatment may alleviate lipotoxicity and thereby promote insulin sensitivity. The hypothesis that a TZD regimen involving repeated treatments of limited duration may suffice for improvements in insulin sensitivity merits further investigation.

## Introduction

The interplay between adipose tissue state and insulin sensitivity has been the subject of numerous studies (for a recent review, see [1], [2])). Adipose tissue grows by two mechanisms: hyperplasia (cell number increase) and hypertrophy (cell size increase). A dysfunction in the availability of lipid storage, normally provided by the growth and development of adipocytes, may be a major contributor to the development of insulin resistance, possibly associated with low-grade chronic inflammation.

Thiazolidinediones are insulin-sensitizing PPAR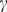 agonists that are known to affect the morphology of adipose tissue. The Zucker fatty rat is a well-characterized model of obesity. Hallakou *et al.* [3] found that pioglitazone (a TZD) treatment of the obese Zucker rat induced an increase in glucose utilization in adipose tissue, a marked increase in weight gain, and stimulated the expression of genes necessary for lipid metabolism. They also found a large increase in small adipocyte recruitment in the retroperitoneal fat pad, as well as a suppression of leptin expression in mature adipocytes. Okuno *et al.* [4] found that troglitazone increased the number of small adipocytes and decreased large adipocytes in obese Zucker rats. de Souza *et al.* [5] found that TZD induced adipose tissue remodeling in female Zucker rats is accompanied by an improvement in insulin sensitivity, and suggested that this is likely a result of lower circulating free fatty acid levels. In human, Boden *et al.* [6] showed that TZDs had multiple effects on patients with Type 2 diabetes, including an increase in small adipocytes in subcutaneous adipose tissue; see also Smith *et al.* [7] and McLaughlin *et al.* [8].

In preliminary experiments (D. Hunt *et al.*, unpublished data), we measured adipose cell-size probability distributions in control and rosiglitazone-treated Zucker fatty rats. These cell-size distributions allow detailed investigation of mechanisms of growth and development of adipose tissue. We observed dynamic changes in cell sizes, appearing to show an acute recruitment of cells to the adipose cell type in treated animals, but the control animals showed dynamic adipose cell-size distributions as well, rendering it difficult to quantify the extent of thiazolidinedione involvement in the dynamic changes. We also observed considerable between–animal variability in these preliminary experiments.

To address TZD effects on the morphology of fat tissue concretely, we obtained cell-size distributions of the inguinal fat pads in six male Zucker (fa/fa) fatty rats by micro-biopsies (Materials and Methods) over a period of 24 days respectively, starting at four weeks of age. This animal model has fat depots that are large enough to allow repeated micro-biopsies to avoid between-animal variability. We developed a Bayesian framework to quantify both the hyperplasia and hypertrophy induced by TZD treatment from these data. Applying the framework, we found that the rosiglitazone treatment leads to an acute hyperplasia within eight days of treatment, and that the treatment leads to an increase in hypertrophy relative to control in intermediate-sized adipocytes.

## Results

### Adipose Cell-Size Distributions

**Figure 1:**
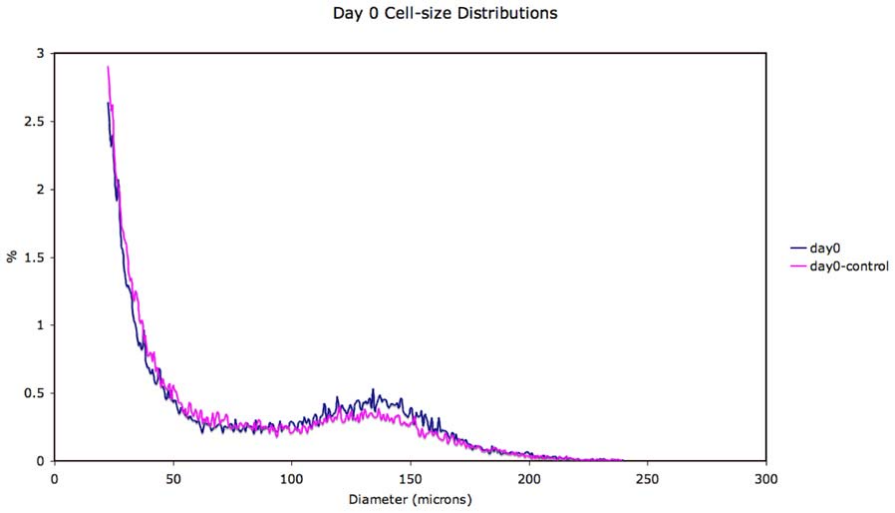
Adipose cell-size probability distributions for treated vs. control animals for day 0.

Representative mean adipose cell-size distributions (see Materials and Methods) comparing treated and control rats on days 0, 16 and 24 are shown in Figs. 1–3. These distributions show dynamics in both control and treated animals. However, a comparison of the changes in the adipose cell-size probability distributions between measured time-points (Figs. 5 and 6) shows a pattern of size increase in treated rats that is distinct from changes in control animals. A heat-map allows a clear visualization of the complete data set showing a linear progression of cell size increase in treated animals (Fig. 4).

**Figure 2 pone-0008196-g002:**
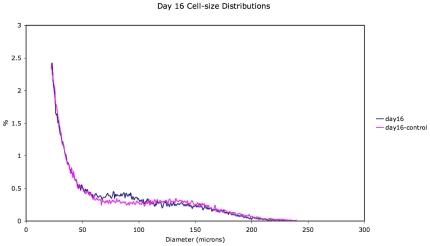
Adipose cell-size probability distributions for treated vs. control animals for day 16.

**Figure 3:**
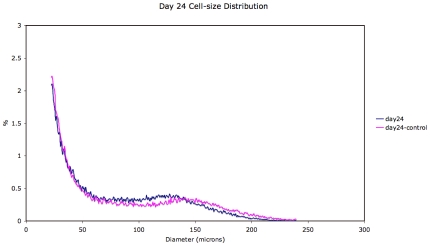
Adipose cell-size probability distributions for treated vs. control animals for day 24.

**Figure 4:**
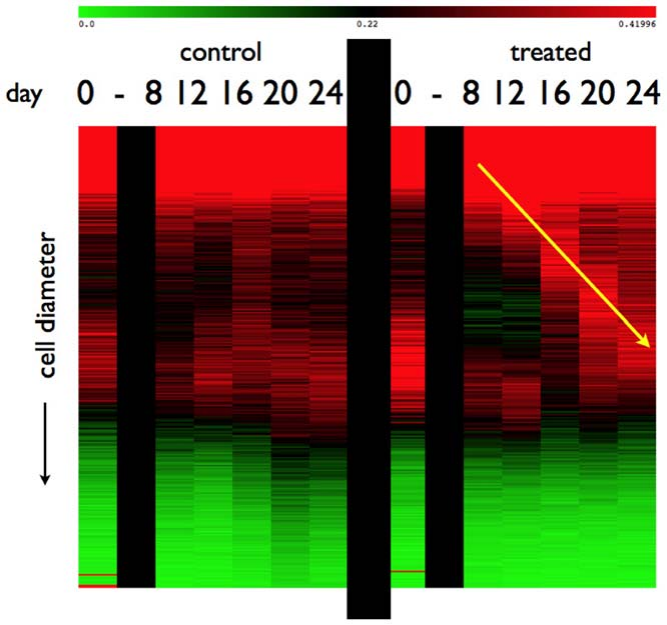
Heat-map of adipose cell-size distributions, showing dynamics in both control and treated animals, with the propagation of a rosiglitazone-induced pulse of adipocytes in treated animals.

**Figure 5:**
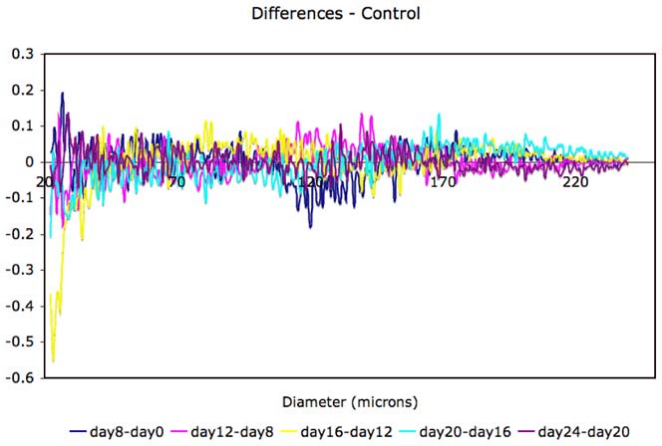
Differences of adipose cell-size probability distributions at successive time points for control animals. The curves show the difference between succeeding day adipose cell-size probability distributions at each cell-size bin 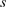
–-for example, the curve labeled (day 12 – day 8) shows Prob(day 12, 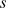) – Prob(day 8, 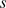).

**Figure 6:**
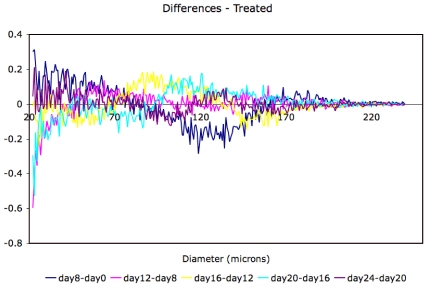
Differences of adipose cell-size probability distributions at successive time points for treated animals. The treated animal differences show a hump of cells moving to larger sizes; this hump is not evident in the contol animals.

### Log-Likelihood of Rate of Cell-Size Increase

**Figure 7:**
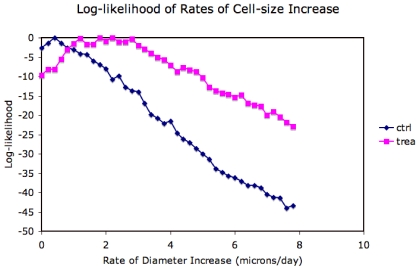
Log-likelihood values for rates of adipose cell diameter increase for control and treated animals. The log-likelihood values for control animals are highest close to 0.44 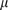
m/day whereas the log-likelihood values are highest around 2 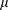
m/day for treated animals.

We evaluated the likelihood of a large range (0 to 7.9 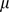
m/day) of rates of adipose cell diameter increase, shown in Fig. 7. The expectation value of the rate of adipose cell diameter increase was 2.02 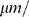
day (st. dev. 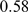 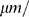
day) for treated animals and 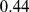 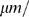
day (st. dev. 0.23 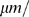
day) for control.

### Adipose Cell Recruitment

**Figure 8:**
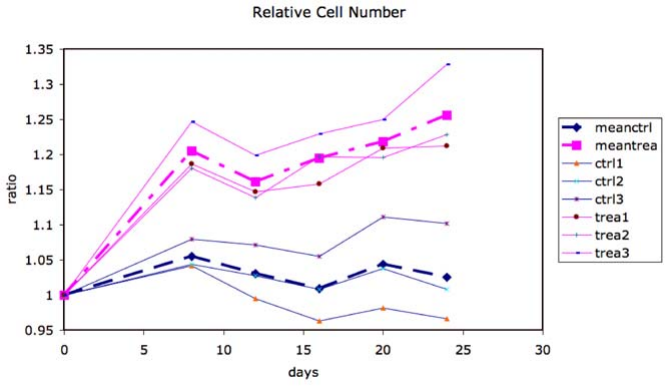
Relative cell number (Total cell number/Total cell number at day 0) as a function of time for control and treated animals.

We evaluated the maximum-likelihood value for the total adipose cell number for all following days of treatment, as a ratio relative to the initial total adipose cell number. The treated animals showed larger relative adipose cell numbers at all time points, as shown in Fig. 8. The treated animals show about 20% more cells by day 8 with a much slower increase at later time points.

### Size-Dependence of Rate of Adipose Cell-Size Increase

**Figure 9:**
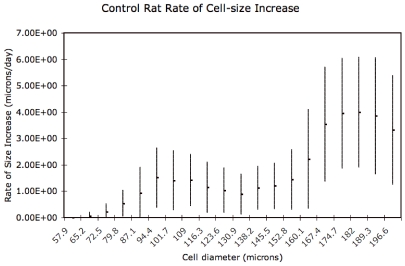
Values and standard deviations of cell-size dependence of rates of cell diameter increase for control animals.

**Figure 10:**
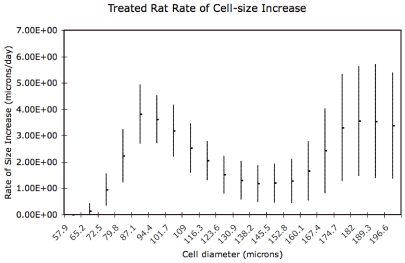
Values and standard deviations of cell-size dependence of rates of cell diameter increase for treated animals. Treated animals show greater rates of cell-diameter increase at intermediate sizes.

We investigated possible size dependence of the rate of adipose cell-size increase by performing our analysis on a moving interval of a hundred cell-size bins. The results are shown in Figs. 9 and 10, suggesting that the change in the rate of adipose cell-size increase connected with treatment primarily affects intermediate (70–120 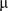
m diameter) adipocytes. The slope of the arrow depicted in Fig. 4 (approximately 4 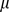
m/day) is consistent with the rate of adipose cell diameter increase at intermediate sizes evident in Fig. 10.

## Discussion

PPAR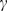 [9] is a nuclear receptor whose agonists include fatty acids and thiazolidinediones. The activation of PPAR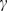 potentially affects a large number of physiological processes, including adipocyte differentiation, with actual activity determined in combination with various co-activators. TZDs improve peripheral insulin sensitivity. Their impact on adipocytes has also been studied [3], [2].

Our results show that TZD treatment leads to more adipocytes, and greater energy uptake by intermediate-sized adipocytes, in the inguinal fat pad of the Zucker fatty rat. We have quantitatively shown that treatment-induced hyperplasia is more or less complete within the first eight days of treatment, with subsequent hyperplasia at a much slower rate. We have also found that an increase in hypertrophy induced by treatment occurs mainly in intermediate-sized adipocytes. This hypertrophy may be related to the greater glucose utilization induced by TZD treatment [3], that in turn may be linked to increased insulin sensitivity in adipocytes [2]. A similar size range for hypertrophy was found in [10], suggesting that the size range for hypertrophy is independent of the stimulus for generating larger adipocytes. On the other hand, the availability of additional adipocytes in a size range capable of additional energy storage due to treatment may itself alleviate hyperglycemia by enabling liponeogenesis.

**Figure 11:**
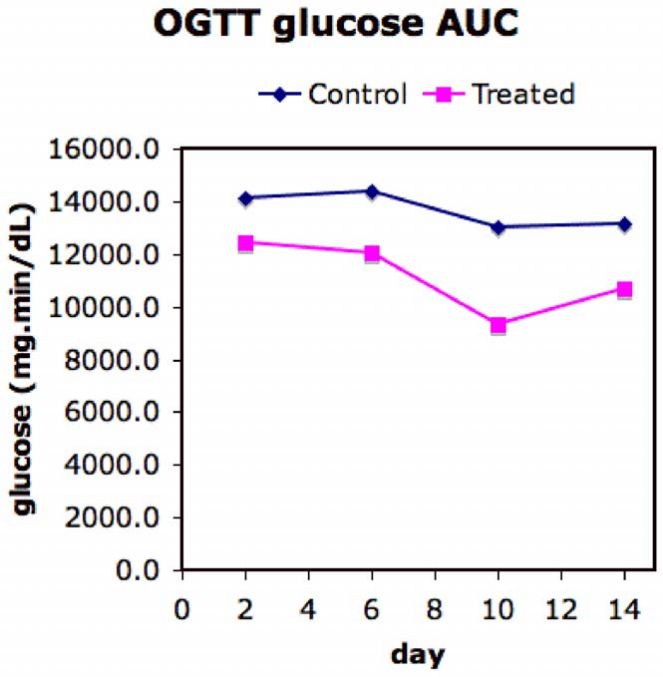
Area-under-the-curve for glucose for Zucker fatty rats, treated with rosiglitazone and control, under an oral glucose tolerance test. The data shown is the mean from three independent experiments with three rats each in control and treated groups. The SEM for the data is 10%.

**Figure 12:**
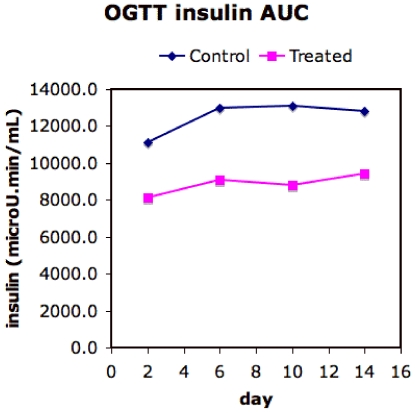
Area-under-the-curve for insulin for Zucker fatty rats, treated with rosiglitazone and control, under an oral glucose tolerance test. The data shown is the mean from three independent experiments with three rats each in control and treated groups. The SEM for the data is 10%.

In data obtained in our laboratory (D. Hunt *et al.*, unpublished) under a protocol in which the rats were killed at days 2, 6, 10 and 14, the insulin sensitizing effects of TZDs appear by day 2 of treatment but there is an decrease in plasma glucose area-under-the-curve (AUC) (Fig. 11) between day 6 and day 10 without a concurrent additional drop in insulin AUC (Fig. 12). This suggests that the availability of additional energy storage in the form of adipocyte recruitment by day 8 (Fig 8) leads to glucose removal from circulation by lipogenesis either in the liver or in the adipose tissue. This may support the hypotheses of de Souza *et al.* [5].

The hypothesis that a TZD regimen involving repeated treatments of limited duration may suffice for improvements in insulin sensitivity merits further investigation.

## Materials and Methods

### Surgical Biopsies

All procedures were carried out in accordance with NIH guidelines for rodent surgery and recovery. Six rats were subjected to the biopsies. Rats were anesthetized with isoflurane. The hair in the inguinal area was clipped and the skin cleaned three times with betadine surgical scrub, followed with an alcohol wipe. For adipogenesis studies in individual animals using surgical biopsies, the sampling procedure involves a small incision (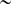 0.1 cm) in the skin just above the inguinal fat depot that lies immediately thereunder, dissection of a 15–20-mg sample of adipose tissue, and closing the incision with stainless steel clips. Clips were removed between 10–17 days. Significant pain and distress were not evident, but animals were closely observed for 2/3 h for potential problems. The inguinal fat depot surrounds the back side of the peritoneum, extending all the way from one hip to the other. Representative samples can be taken all across the fat depot and thus the incisions can be dispersed without giving up the required reproducibility. The biopsies were done on days 0, 8, 12, 16, 20 and 24; on each day for each rat, duplicate biopsies were obtained, one each from similar sites on each side of the midline.

### Treatment

The rats were randomized into two equal groups: three animals treated with vehicle (water) and three treated with rosiglitazone. The rosiglitazone group was dosed (3 mg/kg) daily via oral gavage for the duration of the experiment (24 days).

### Measurement of Adipose Cell-Size

Adipose cell-size distribution was assessed using a Beckman Coulter Multisizer III as previously described [11]. Briefly, 15–20 mg of fat were immediately fixed in osmium tetroxide [12] and incubated in a water bath at 37

C for 48 h. Adipose cell-size was then determined by a Beckman Coulter Multisizer III with a 400 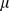
m aperture. The range of cell-sizes that can effectively be measured using this aperture is 20–240 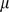
m. The instrument was set to count 6,000 particles, and the fixed-cell suspension was diluted so that coincident counting was 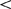
10%. After collection of pulse sizes, the data were expressed as particle diameters and displayed as histograms of counts against diameter using linear bins and a linear scale for the cell diameter. Cell-size distribution was measured four times from each biopsy, divided into two separate suspensions that were each counted twice.

### Bayesian Analysis of Data

The aim of our analysis was to analyze longitudinal adipose cell-size distributions to quantitatively characterize hypertrophy and hyperplasia of adipose tissue in Zucker fatty rats induced by rosiglitazone. We introduced a set of models in which cell diameter grew linearly at differing rates, ranging from 0 to 7.9 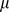
m/day, in increments of 0.1 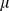
m/day. For a given model of rate 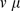
m/day, we expected that cells at diameter 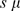
m will have increased in size to 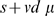
m after 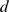 days. Given that the data consists of probability distributions of cell-sizes but not absolute cell numbers, we also had to infer a change in the absolute cell number, since we were comparing absolute cell numbers in such a comparison of cell-size change as a function of 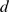 days. Thus, we introduced a relative cell number parameter 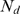 for each day after day 0 of the experiment, with 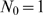. We took the log of the probability of the data, given the model, to be the sum over bin sizes, 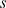, and pairs of successive time points, 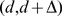, of
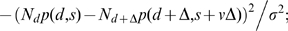
(1)here, 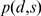 is the adipose cell-size probability distribution at diameter 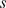 on day 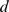.

We marginalized over 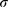 and 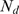 using a parallel tempered Monte Carlo procedure given by Gregory [13], with uninformative priors for all variables. The initial value of 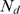 was taken to be 1 for all days, and the initial value of 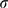 was taken to be 0.05, the estimated measurement uncertainty in the data. The Markov Chain Monte Carlo carried out equilibration for 3000 steps (with a mean fractional parameter step size of 0.05) and then marginalized over the next 300 steps (with a mean fractional parameter step size of 0.01). During the equilibration time, the parallel tempering procedure switched the ten evenly spaced temperatures with a probability of 0.05, corresponding to about once every twenty steps. After equilibration, the parallel tempering steps were all started from the same initial value, the maximum likelihood value found during the equilibration phase of the Monte Carlo procedure, and did not undergo any further switching between temperatures to ensure that the integration-over-temperatures interpretation of parallel tempering was valid.

To determine the size-dependence of the rate of cell-diameter increase, we carried out the entire analysis as described above on moving windows of a hundred cell-size bins (corresponding to a window of 60 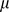
m). The probability-weighted mean velocity for the moving cell-size windows is plotted in Figs. 9 and 10, with the probability-weighted standard deviations as the uncertainty bars.

All analysis was performed in XLispStat [14].
